# Molecular characterization and transcriptional response to TiO_2_–GO nanomaterial exposure of two molt-related genes in the juvenile prawn, *Macrobrachium rosenbergii*

**DOI:** 10.1038/s41598-023-37626-8

**Published:** 2023-06-27

**Authors:** Ziqi Guo, Likun Xu, Wei Wang, Wei Chen, Chunyan Ma, Fengying Zhang, Lingbo Ma, Zhiqiang Liu, Keyi Ma

**Affiliations:** 1 Key Laboratory of East China Sea Fishery Resources Exploitation, Ministry of Agriculture and Rural Affairs, East China Sea Fisheries Research Institute, Chinese Academy of Fishery Sciences, No.300 Jungong Road, Yangpu Area, Shanghai, 200090 People’s Republic of China; 2 College of Fisheries and Life Science, Shanghai Ocean University, Pudong New Area, Shanghai, 201306 People’s Republic of China

**Keywords:** Molecular biology, Environmental sciences

## Abstract

In recent years, with the widespread use of TiO_2_–GO nanocomposite in industry, especially in the remediation of water environments, its toxic effects on aquatic organisms have received increasing attention. As molting is extremely important for crustaceans in their growth, in this study, we cloned the full-length cDNA sequences of two key genes related to molting, *nuclear hormone receptor E75* (*E75*) and *nuclear hormone receptor HR3* (*HR3*), in *Macrobrachium rosenbergii*, examined the gene expression profile, and investigated their toxicological effects on crustacean molting through nanomaterial exposure. The amino acid sequences for E75 and HR3 were respectively determined to encode 1138 and 363 acid residues. Sequence analysis showed that both E75 and HR3 contain a HOLI domain, with the *E75* of *M. rosenbergii* being more closely related to the *E75* of *Palaemon carinicauda*. These two genes were expressed at the highest levels in muscle, followed by hepatopancreas. The results showed that the expressions of *E75* and *HR3* in hepatopancreas and muscle tissues were significantly decreased after exposure to 0.1 mg/L of TiO_2_–GO composite nanoparticles (*P* < 0.05). This study will serve as a foundation for subsequent research into the evaluation of nanomaterial toxicity on crustacean species.

## Introduction

The use of titanium dioxide nanomaterials (nTiO_2_) has increased significantly in recent years^1^. Piccinno documented the estimated worldwide production of nTiO_2_ at approximately 5000 t/year in 2006–2010 and 10,000 t/year in 2011–2014, predicting an even higher production of this nanomaterial by 2025^2^. This impressive increase is mainly due to the demand for this metal oxide in the paper, paint, coating, cosmetics, food, and plastic industries. In industrial application, the photocatalytic efficiency of titanium dioxide is usually improved by hybridization with other components, such as graphene oxide (GO)^3^. Previous studies have shown that TiO_2_–GO achieved both higher separability and good photocatalytic activity^4^. Due to its excellent performance, it is used in a wide range of industrial applications, especially in environmental remediation (e.g., pollutant degradation in water).

As nanomaterials continue to enter the aquatic environment, there is widespread concern about their toxicity to the aquatic environment and to aquatic organisms. Direct evidence for the release of synthetic nanomaterials from urban sources into the aquatic environment has already been reported^5^. In particular, freshwater and coastal environments, due to their proximity to human population and industrial centers, face a notable risk from nanomaterials released through wastewater discharges^6^. Hence, while nanomaterials persist in marine ecosystems, they are also predominantly found in freshwater lakes, streams and rivers, which are important entry points into estuaries and ultimately into the marine environment. In terms of their interaction with living things, studies have shown that nanomaterials are accumulated in the digestive system and can even enter the circulatory system^7^. Much knowledge already exists on the impacts of TiO_2_ nanoparticles on aquatic organisms such as bacteria, algae, crustaceans, nematodes as well as fishes^8^. However, few studies have examined the toxic effects of the TiO_2_–GO composite nanomaterials on aquatic organisms.

The giant river prawn (*Macrobrachium rosenbergii*), belonging to the Arthropod subphylum Crustacea, is one of the most important aquaculture shrimps worldwide. It has an extremely broad distribution worldwide, in the sea, in estuaries and in freshwater. It is an important aquaculture species in many Asia–Pacific countries due to its rapid growth, wide adaptability, high disease resistance and high economic value^9^. As composite nanomaterial contamination has become a major issue in aquaculture systems, including *M. rosenbergii* breeding, related studies are beginning to appear in the literature. For example, studies on the effects of TiO_2_ composites on aspects such as growth, digestive enzyme activity, oxidative stress, immune enzyme activity and transcriptomics in *M. rosenbergii* have been done^10,11^. On the other hand, before the current study, the effects of composite nanomaterials on prawn molting had yet to be examined.

Previous studies have shown that exposure to nanomaterials has a deleterious effect on the molt, or ecdysis, of *Daphnia pulex* and its physiological processes, with an inhibitory effect on the molt^12^.To investigate the effect of nanomaterial exposure on the ecdysis of *M. rosenbergii*, two genes associated with the ecdysis process were selected in the current study, namely the *nuclear receptors HR3* (group NR1F) and *nuclear receptors E75* (group NR1D), which are two important transcriptional regulators in insect ecdysteroid signaling cascades^13^. Through these cascades, ecdysis is induced by molting hormone ecdysones, generally called ecdysteroids^14^. Firstly, ecdysone is metabolized by ecdysone 20-monooxygenase (E20MO; 20-hydroxylase) which mediates the conversion of ecdysone (E) to the active molting hormone, 20-hydroxyecdysone (20E)^13^. The nuclear receptor superfamily members, ecdysone receptor (EcR) and retinoid X receptor (RXR), combine to produce a heterodimeric receptor that is delivered by the hemolymph to the target cells. The EcR/RXR heterodimers can bind to hormone response elements and control the transcription of genes encoding transcription factors, such as *HR3* and *E75*^15^. These transcription factors then control other downstream transcriptional cascades that result in various enzymatic activities correlating to the breakdown of both the old cuticle and the synthesis of the new cuticle^16^. In particular, HR3 activates target downstream genes in the signaling pathway while *E75* is most often known as a negative regulator of *HR3* transcriptional activity^17^. In previous studies, the *E75* and *HR3* genes have been cloned in the crustacean *Daphnia*^18^. Since they are found in crustaceans, it is conceivable that they play a significant part in the signaling of ecdysteroids in these animals, just as they do in insects.

In this study, for the first time, the expression of *nuclear hormone receptor HR3* and *nuclear hormone receptor E75* as indicators for the ecdysis process were examined in juvenile *M. rosenbergii* exposed to various concentrations of TiO_2_–GO composite nanomaterials. This research offers a theoretical framework for understanding how nanomaterials affect the ecdysis process in aquatic crustaceans, and may be utilized to further investigate the molecular impacts of composite nanomaterials on these organisms.

## Materials and methods

### M. rosenbergii culture

*Macrobrachium rosenbergii* juveniles were acclimated in fresh water for two weeks so that they would acclimatize to the experimental environment. Water temperatures throughout the acclimation rearing varied from 24 to 26 °C, the pH was kept between 7.5 and 8.0, and the dissolved oxygen concentrations were kept above 6 mg/L. For the subsequent experiment, we randomly selected approximately 900 healthy prawns, of uniform size and initial body length of 0.68 ± 0.12 cm, and evenly distributed them into nine separate net tanks, each holding one cubic meter of water. Thereafter, these prawns were given commercial feed twice daily for 45 days, and also subjected to the following procedures.

### TiO_2_–GO nanocomposite synthesize and exposure

TiO_2_ was purchased from Innochem Co., Ltd (Beijing, China). Graphene oxide (GO) was synthesized by the modified Hummers’ method^19^. The TiO_2_–GO was obtained via a hydrothermal method based on Rajamathi’s work with modifications^20^. Briefly, 4 mg GO was dissolved in a mixed solution of 40 mL of ultrapure water and 20 mL of ethanol by ultrasonic treatment for 1 h, and 0.4 g of TiO_2_ was added to the obtained GO solution and ultrasonic treatment another 2 h to get a homogeneous suspension. The suspension was then placed in a 100 mL Teflon sealed autoclave and maintained at 120 °C for 3 h. Finally, the obtained product was washed several times with deionized water and dried at room temperature. Aqueous suspensions of TiO_2_–GO nanocomposite were made into the colloids for exposure experiments.

### Experimental design for chronic nanomaterials exposure

The nine net tanks in which we placed the experimental *M. rosenbergii* were divided into three groups of three tanks each. The prawns in one group were used as controls, whereas those in the other two groups were exposed, respectively, to two nominal concentrations of TiO_2_–GO nanomaterial suspension (0.1 and 0.5 mg/L), based on related literature^21^. Every two days, newly prepared TiO_2_–GO nanomaterials was used in an exchange of 1/2 to maintain the treatment concentrations. Based on this design, each group would have three replicates, each comprised of 100 prawns.

### RNA extraction and full-length cDNA cloning

After 45 days, total RNA was extracted from the prawns using RNAiso Plus (TaKaRa, Tokyo, Japan) according to the manufacturer's protocol. The concentration of total RNA of each sample was quantitated using Nanodrop 2000c (Thermo Scientific, Waltham, MA, USA). Then, mRNA was isolated from the total RNA and the cDNA was synthesized.

The *HR3* and *E75* gene fragments of *M. rosenbergii* were obtained and verified from the transcriptome database in our laboratory (unpublished). BLAST comparison and open reading frame (ORF) analyses showed that the gene fragments possessed 5′-terminal sequences. RACE 3′-terminal primers were design using Primer 5 (Table 1).

**Table 1:** Sequences of primers used in this study.

Gene	Sequence (5′-3′)
*E75 F1*	CCTCCGATCCGATCATAACGC
*E75 F2*	GTCGCAGCATCCAGCAGAAGCTT
*HR3 F1*	CGCCCACATGGATACCTGCGAATT
*HR3 F2*	TGATGCCCGCACCAATACAATG
*qE75 F*	GGCGGCAGGTACAAAGAGA
*qE75 R*	TAGGCTAGTATTGCGGGGA
*qHR3 F*	GCGTGGACAGGAGGAAATC
*qHR3 R*	AGTGACAAATACGGAAGAACC
*β-actin F*	GTAGGTGGTCTCGTGAATGCC
*β-actin R*	TCGTGCGTGACATCAAGGAAA

The expression products of *E75* and *HR3*'s cDNA end amplification stages were accomplished using the 3'-RACE Kit (Sangon Biotech Co., Ltd., Shanghai, China) in accordance with the manufacturer’s instructions. The outer forward primers E75F1 and HR3F1 and the nested primers E75F2 and HR3F2 were designed from known sequences in the transcriptome database, and the full-length 3′ ends of *E75* and *HR3* were further amplified with adaptor primers. Touchdown PCR was performed under the following conditions: 10 cycles of denaturation at 94 °C for 40 s, 25 cycles of annealing at 63 °C for 40 s and cool down at a rate of 0.5 °C per cycle, and a final cycle of annealing at 58 °C for 40 s and extension at 72 °C for 1 min. All amplified products were purified using a DNA purification kit (TaKaRa, Tokyo, Japan). And the RACE-PCR products were then used in a nested PCR, which was based on^22^. Then, the amplified RACE-PCR products were examined for band clarity and fragment length using agarose gel electrophoresis.

In order to assess the accuracy of the cDNA sequence, the sequencing results were compared using BLAST in the NCBI database to identify the amino acid sequence homology of the same gene in related species. The acquired 5′ and 3′ terminal and intermediate sequences were spliced using DNAMan software to produce the full-length *E75* and *HR3* cDNA sequences after successful comparison and detection.

### Bioinformatics analysis

In this experiment, ORF Finder was used to predict the ORF interval. ExPASy-ProtScale (https://web.expasy.org/protscale/) was used to examine the hydrophobic regions of proteins, while BLASTP was utilized to analyze homologous proteins. ExPASy-ProtParam (https://web.expasy.org/compute_pi/) was used to study the amino acid composition, relative molecular mass, and isoelectric point. Protein secondary and tertiary structures were evaluated, respectively, using PSIRED Protein Structure Prediction Sever and SWISS-MODEL Sever, while protein domains were examined using SMART. MEGA11 was applied to create phylogenetic trees in order to compare gene sequence homologies. For the construction of phylogenetic trees, the evolutionary history was inferred using the neighbor-joining technique. The NCBI database served as the foundation for the species' amino acid sequence alignment.

### Real-time quantitative PCR analysis

RNA was collected from prawn tissues including the hepatopancreas, gill, brain, muscle, heart, and eyestalk in order to conduct the expression profiling of *E75* and *HR3*. The primers qHR3F, qHR3R, qE75F, and qE75R were designed using the Primer Premier 5.0 program and the primer sequences are summarized in Table 1. Additionally, the housekeeping gene *β*-*actin* was selected as the internal gene. CFX96™ Thermal Cycler (BioRad, Hercules, CA, USA) was employed to perform qRT-PCR. PCR amplification program designed according to the specifications of NovoStart^®^SYBR qPCR SuperMix Plus (Novoprotein, China). The triplicate fluorescence intensities were measured in terms of the crossing-point (Ct) values. The supplemental material contains more information about this procedure.

### Statistical analysis

SPSS 23.0 was used for statistical analysis of the experimental data, and the results were expressed as mean ± standard error of mean (SEM). Relative mRNA levels of target genes were analyzed using the 2^−ΔΔCt^ method. Significance of differences was tested using one-way analysis of variance (ANOVA) and *P* < 0.05 was accepted as the threshold of significant difference.

## Results

### TiO_2_–GO nanocomposite characterization

The fourier transform infrared spectroscopy (FTIR) spectra of TiO_2_–GO showed that the oxygenated functional groups demonstrated high absorption peak intensity in the range of 1000–2000 cm^−1^. Besides, the strong peaks in the range of 400–1000 cm^−1^ could be attributed to a combination of the Ti–O–Ti and Ti–O–C stretching vibrations. FTIR data and TEM results were reported in^10^.

### Full-length sequence analysis

**Figure 1:**
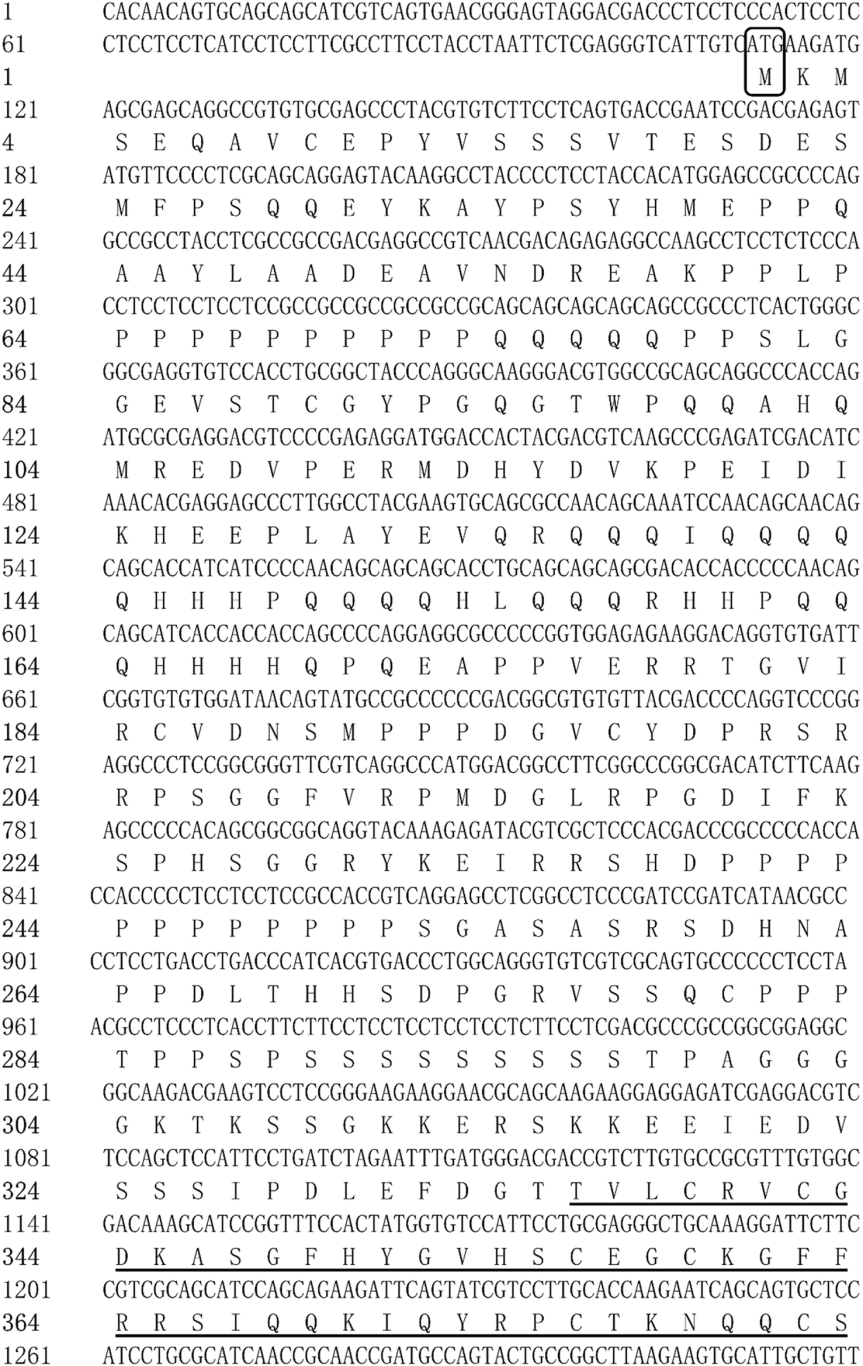

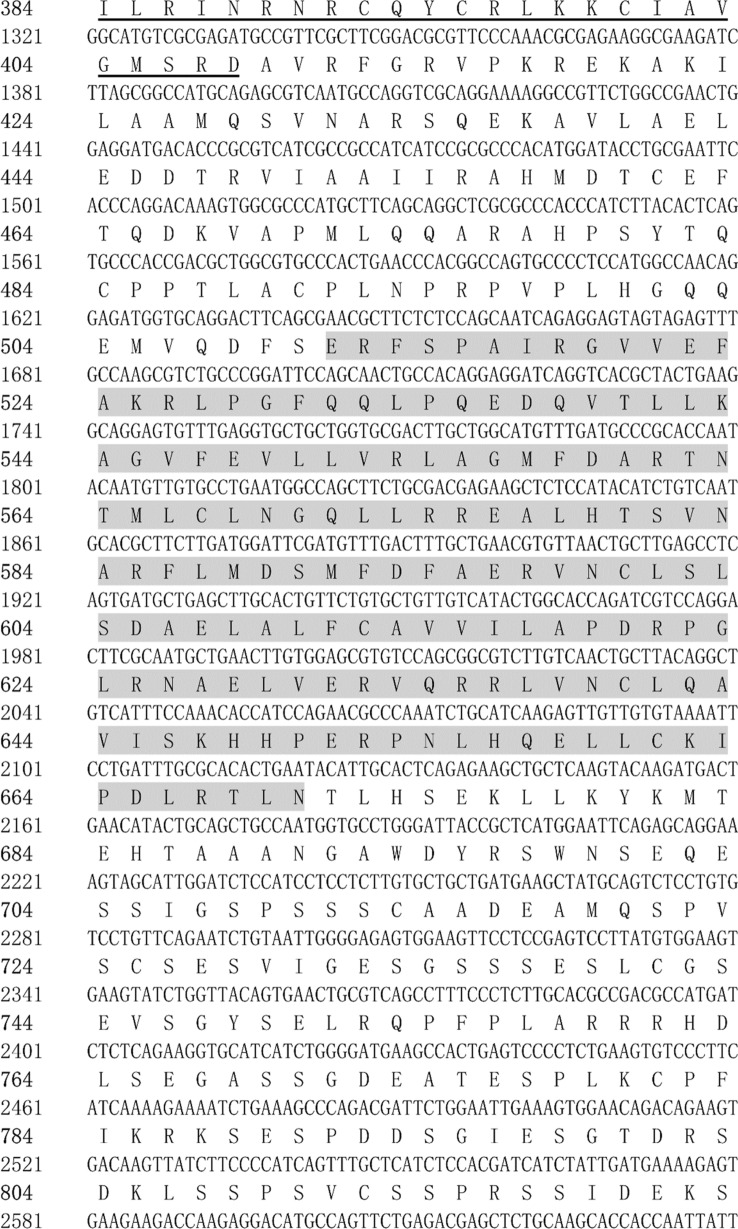

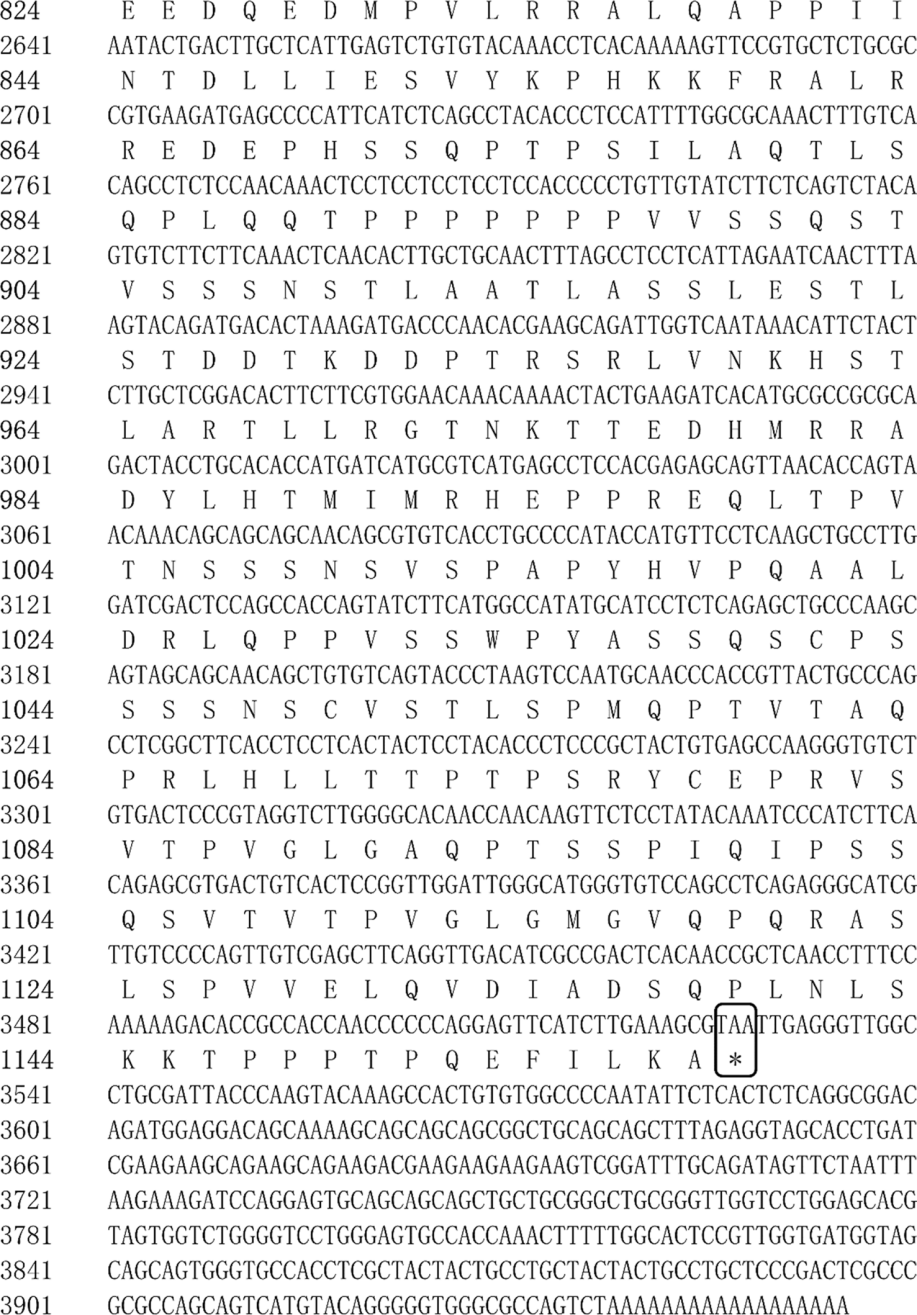
Full-length cDNA cloning results of *E75* from *M. rosenbergii*. The start codon (ATG) and end codon (TAA) are highlighted using black boxes. The ZnF_C4 domain is shown by a black line. The HOLI domain is indicated by a gray box.

**Figure 2:**
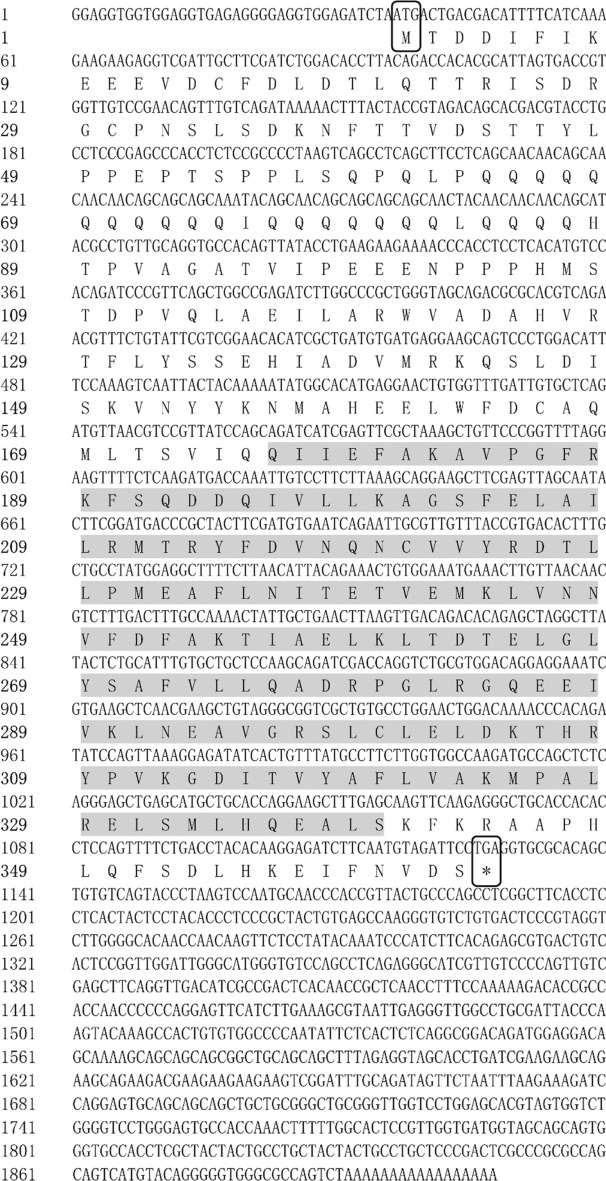
Full-length cDNA cloning results of *HR3* from *M. rosenbergii*. The start codon (ATG) and end codon (TGA) are highlighted using black boxes. The HOLI domain is indicated by a gray box.

The full-length cDNA sequences of *E75* (GenBank accession No. OQ626397) and *HR3* (No. OQ626398) were identified to be 3955 and 1907 bp long, respectively. The full-length cDNA sequence of E75 included a 111 bp 5′-untranslated region (5′-UTR), a 3417 bp ORF region encoding 1138 amino acids, a 427 bp 3′-UTR, and a poly-A tail at the 3′ end (Fig. 1). The full-length cDNA sequence of HR3 included a 36 bp 5′-UTR, and a 1092 bp ORF region encoding 363 amino acids, a 779 bp 3′-UTR, a HOLI region, and a poly-A tail at the 3′ end (Fig. 2).

**Figure 3:**
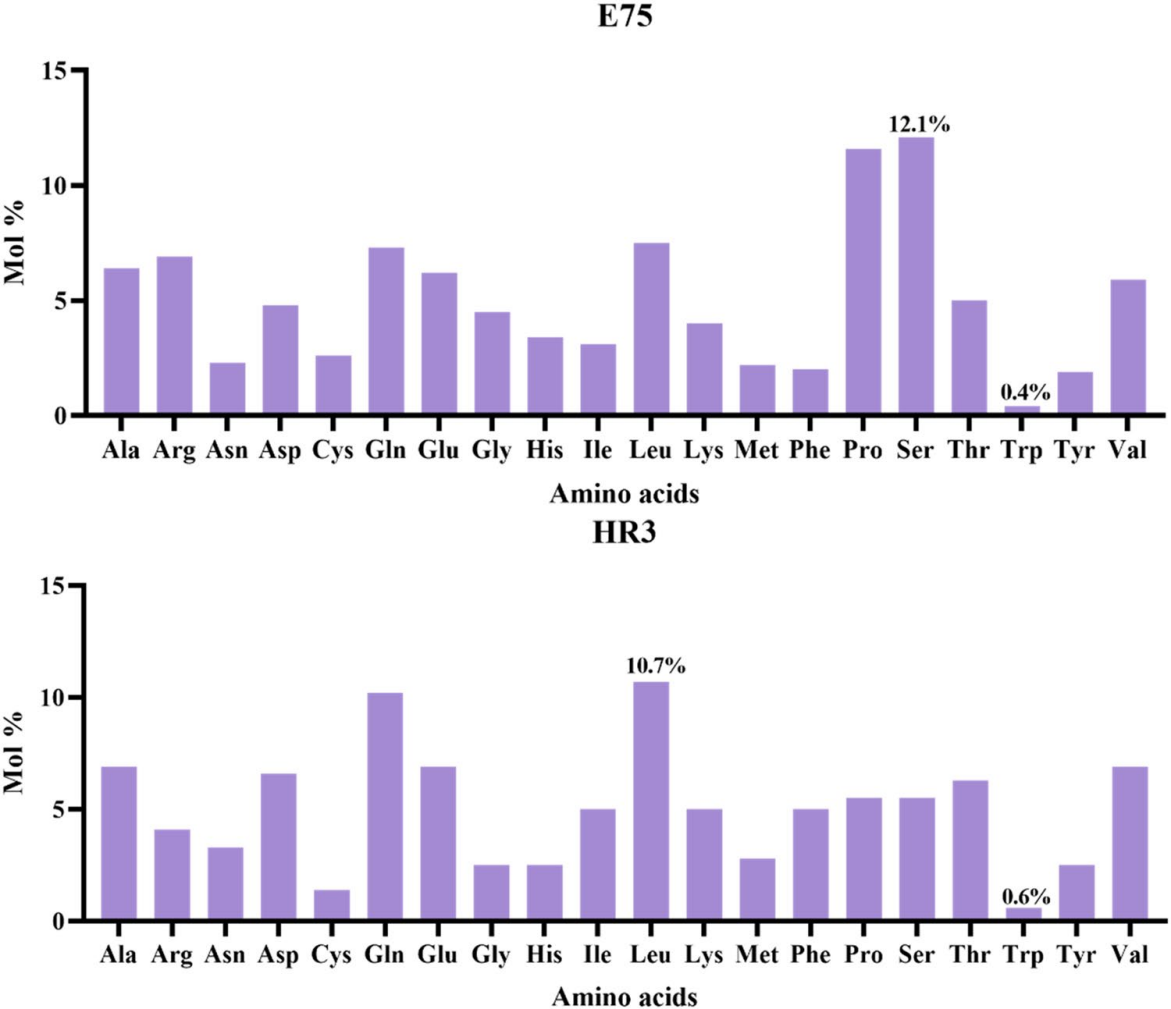
Composition of amino acids in the E75 and HR3 proteins in *M. rosenbergii.*

**Figure 4:**
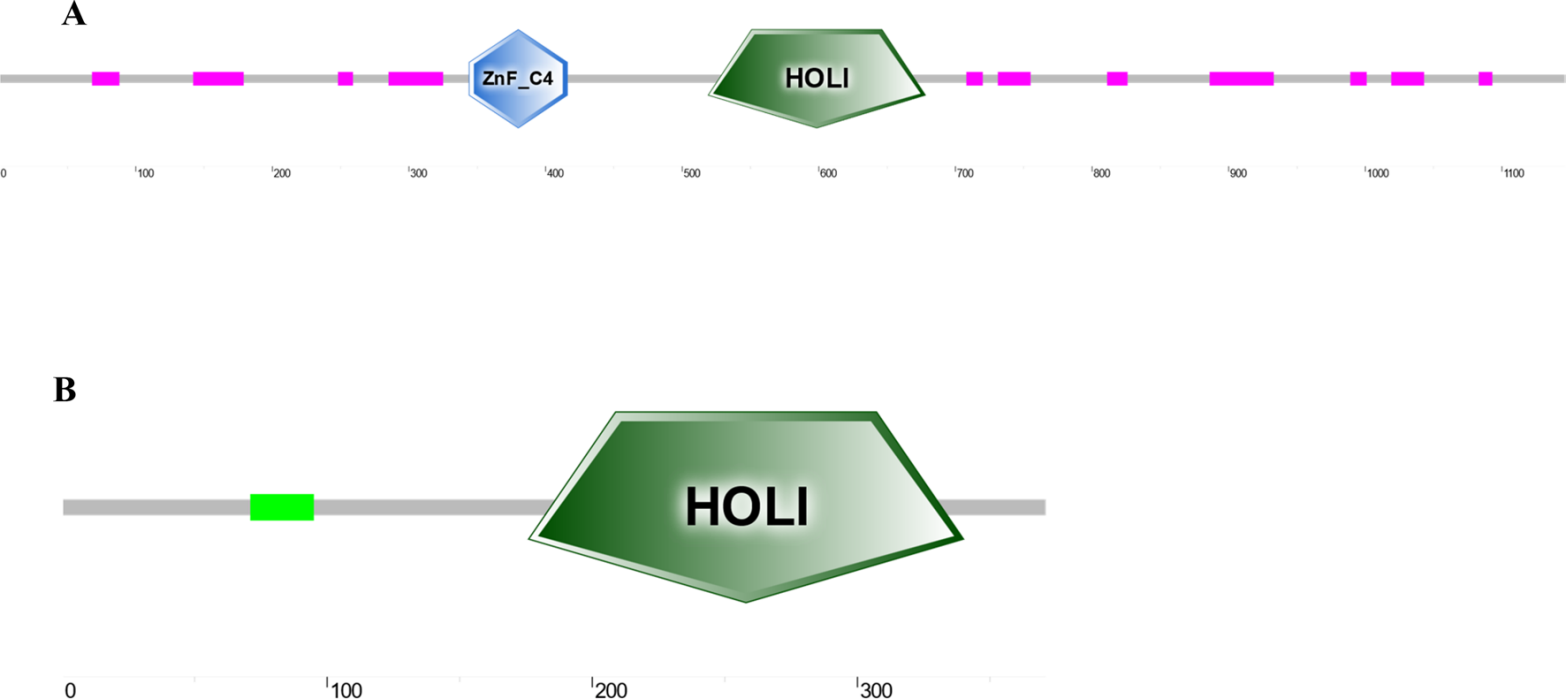
Domain analysis of *E75* (**A**) and *HR3* (**B**) in *M. rosenbergii.*

The molecular weight (Mw) of the *E75* protein was 125.3 kD, and the theoretical isoelectric point was 7.09. According to the anticipated full-length amino acid composition of *E75*, serine (Ser) had the highest occurrence (12.1%), while tryptophan (Trp) had the lowest level (0.4%) (Fig. 3). The Mw of the *HR3* protein was 41.5 kD, and the theoretical isoelectric point was 5.01. Among the amino acids, 125 were positively charged (Arg + Lys) and 123 were negatively charged (Asp + Glu). The aliphatic index is 64.61. The full-length cDNA contained 11 low complexity regions, a ZnF_C4 domain and a HOLI domain. The HOLI domain, starting at Glu^511^ and ending at Asn^670^, was a typical structural organization of the prototypical nuclear receptors (Fig. 4A). The anticipated outcome revealed that the *E75* protein's secondary structure had plenty of α-helix and β sheets (Fig. S1).

The prediction results of the full-length amino acid composition of *HR3* showed that the occurrence of Leucine (Leu) was the highest (10.9%), and that of tryptophan (Trp) was the lowest (1.9%) (Fig. 3). Among all of the amino acids, 49 were positively charged (Arg + Lys) and 33 were negatively charged (Asp + Glu). The aliphatic index of *HR3* is 88.10. The full-length cDNA cloning results showed that there were a coiled-coil region and a HOLI domain starting at Gln^176^ and ending at Ser^340^ (Fig. 4B). The anticipated outcome revealed that the *HR3* protein's secondary structure had plenty of α-helix and β sheets (Fig. S2).

### Phylogenetic tree construction

Phylogenetic tree analysis based on the neighbor-joining technique method shows that the *E75* and *HR3* of *M. rosenbergii* also have a high degree of phylogenetic proximity to corresponding genes in other crustaceans and some insects. Bootstrap analysis with 1000 replicates for each branch position was used to assess support for nodes in the tree. The ecdysteroid receptor gene of *Daphnia magna* was chosen as the outgroup. BLAST alignments of the *E75* and *HR3* sequences revealed 72–84% identity with a number of insects (viz: *Habropoda laboriosa*, *Chelonus insularis* and *Psylliodes chrysocephala*).

**Figure 5:**
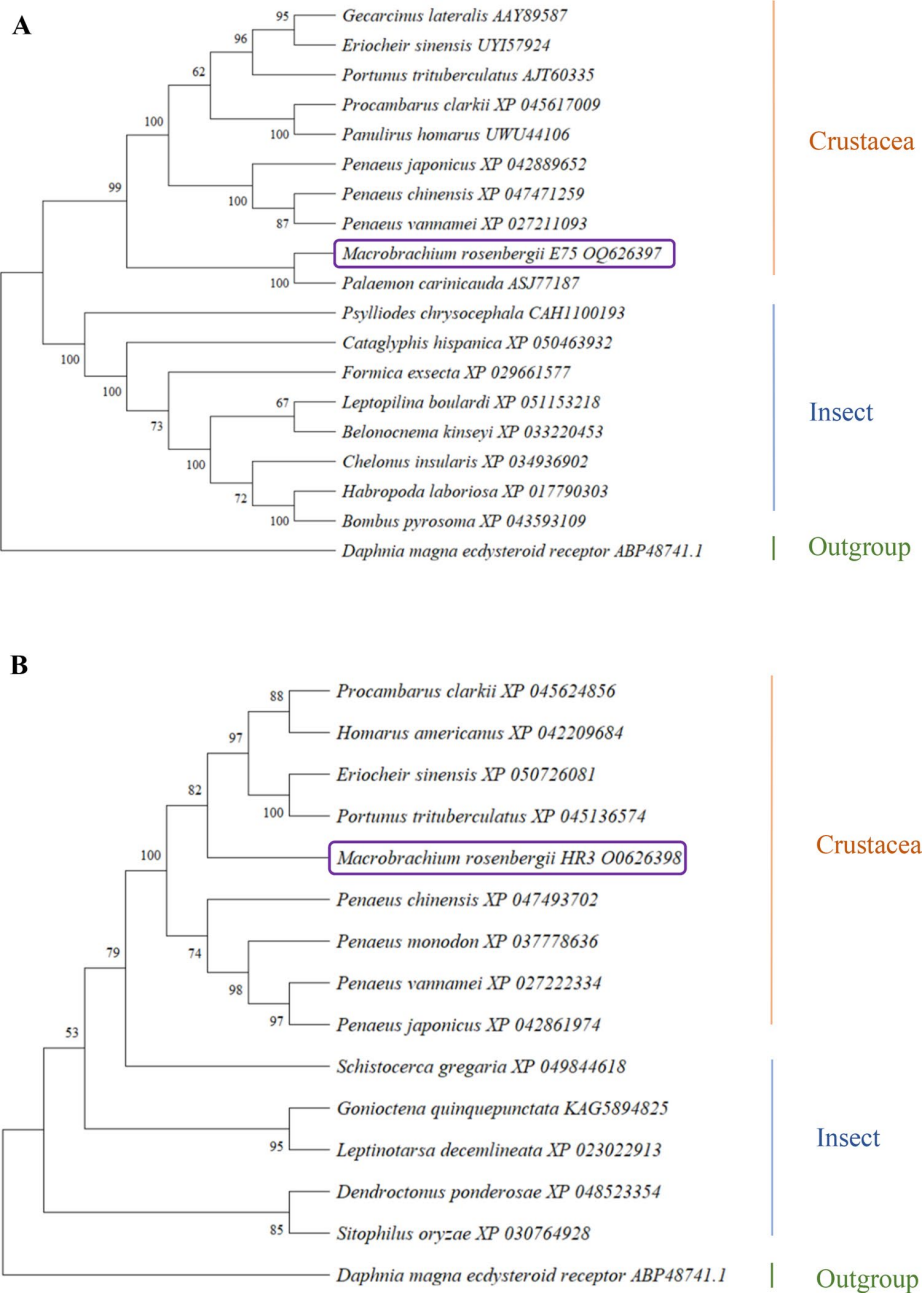
Phylogenetic trees of E75 and HR3 constructed according to the neighbour-joining method of MEGA 11. (**A**) is the phylogenetic tree of *E75*; (**B**) is the phylogenetic tree of *HR3*.

Aside from the outgroup, the phylogenetic tree of *E75* splits into two clusters comprised, respectively, of insect and crustacean sequences (Fig. 5A). According to the phylogenetic tree, the branch most closely related to *M. rosenbergii*, as inferred from that the amino acid sequence of the *E75* protein, is *Palaemon carinicauda*.

The phylogenetic tree of *HR3,* based on the amino acid sequences of the *HR3* proteins, is shown in Fig. 5B. For this tree, once again, all the crustaceans (aside from the outgroup) form one cluster. On the other hand, some insects appear more closely related to the above cluster than to the other insects. Among the insects, *Schistocerca gregaria* is the branch having the closest relationship with *M. rosenbergii*. Within the crustacean cluster, *M. rosenbergii* is the sister branch to the sub-cluster comprised of *Procambarus clarkii, Homarus americanus*, *Portumnus trituberculatus* and *Eriocheir sinensis*.

### Tertiary structure of protein

Two crustacean species, along with *M. rosenbergii,* were selected for analysis of the tertiary structure of the proteins. The three-dimensional space-filling structure of E75 from *M. rosenbergii* (Mr-E75), *Penaeus vannamei* (Pv-E75) and *Procambarus clarkii* (Pc-E75) was established based on a template from the crystal structure of *Homo sapiens* Retinoic acid receptor RXR-alpha (PDBe 4nqa.1. A.) (Fig. 5). The predicted three-dimensional structure of *E75* showed that *E75* had more than ten α-spirals, some β-strands, and a random coil connecting them. The similarity between the template and the three sequences ranged from 30.67 to 30.79%, indicating that these E75 isoforms might have a similar structure. QEMAN (Qualitative Model Energy Analysis) was ˃ − 4.82, and GMQE (Global Model Quality Estimation) was ˃ 0.17 (Table S1).

**Figure 6:**
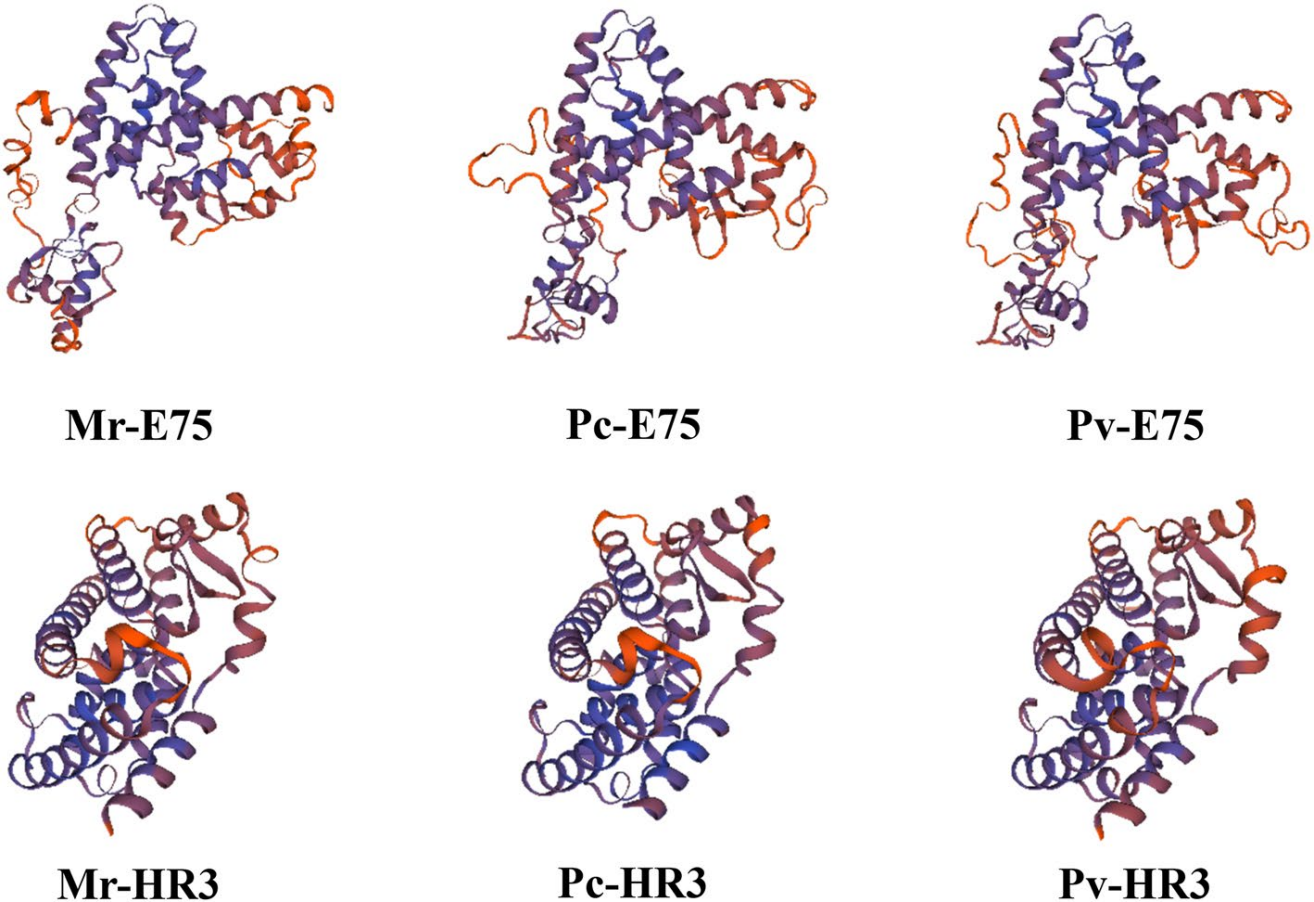
The predicted spatial structures of Mr-E75, Pc-E75, Pv-E75, Mr-HR3, Pc-HR3, and Pv-HR3.

The tertiary structure of HR3 from *M. rosenbergii* (Mr-HR3), *Penaeus vannamei* (Pv-HR3) and *Procambarus clarkii* (Pc-HR3) was established based on the crystal structure of nuclear receptor ROR-alpha (PDBe 4s15) (Fig. 6). The predicted three-dimensional structure of *HR3* showed that *HR3* had a lot of α-spirals and some β-strands, which were connected by a random coil. The sequence identity between Mr-HR3, Pv-HR3, Pc-HR3 and the template ranged from 34.44 to 34.73%. QEMAN was ˃ − 1.89 and GMQE was ˃ 0.36, indicating the high quality of these results. A considerable degree of evolutionary resemblance between the three species was shown by the sequence similarities and the analysis of the spatial structures.

### Expression analysis in different tissues

**Figure 7:**
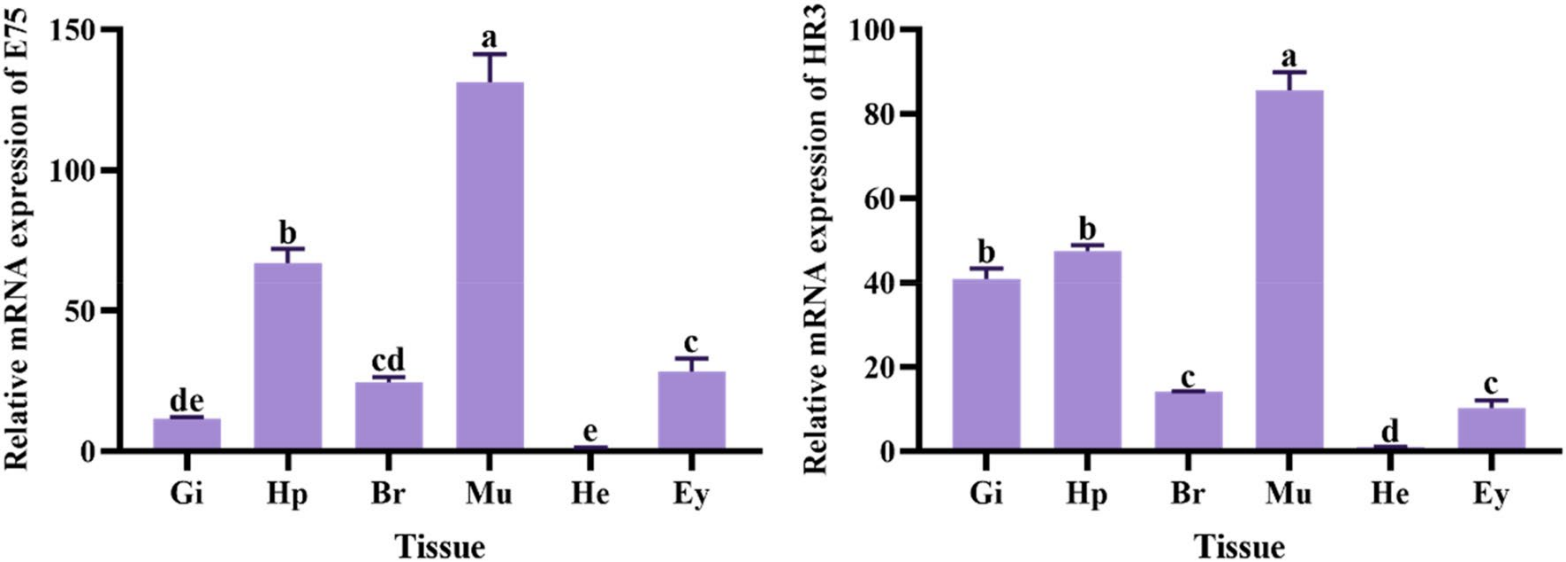
Relative expression of the *E75* gene and *HR3* gene in different tissues of *M. rosenbergii*. The abscissa abbreviations stand for: Gi-Gill, Hp-Hepatopancreas, Br-Brain, Mu-Muscle, He-Heart, Ey-Eyestalk. Data are presented as mean + SEM. Different letters above the bars indicate significant differences (*P* < 0.05).

Quantitative fluorescence analysis was carried out on the expression of the two genes from different tissues of *M. rosenbergii* (Fig. 7). The highest level of *E75* expression in juvenile prawns was in muscle, followed by hepatopancreas. There were no significantly different expression levels in brain, gill and eye (*P* > 0.05). For *HR3*, the highest expression level in juvenile prawns was in muscle, followed by hepatopancreas and gill. There was no significant difference between brain and eye (*P* > 0.05). In these tissues, the expression of both *E75* and *HR3* was significantly lower in the heart than in other tissues (*P* < 0.05).

### Expression analysis in the presence of TiO_2_–GO nanomaterials

**Figure 8:**
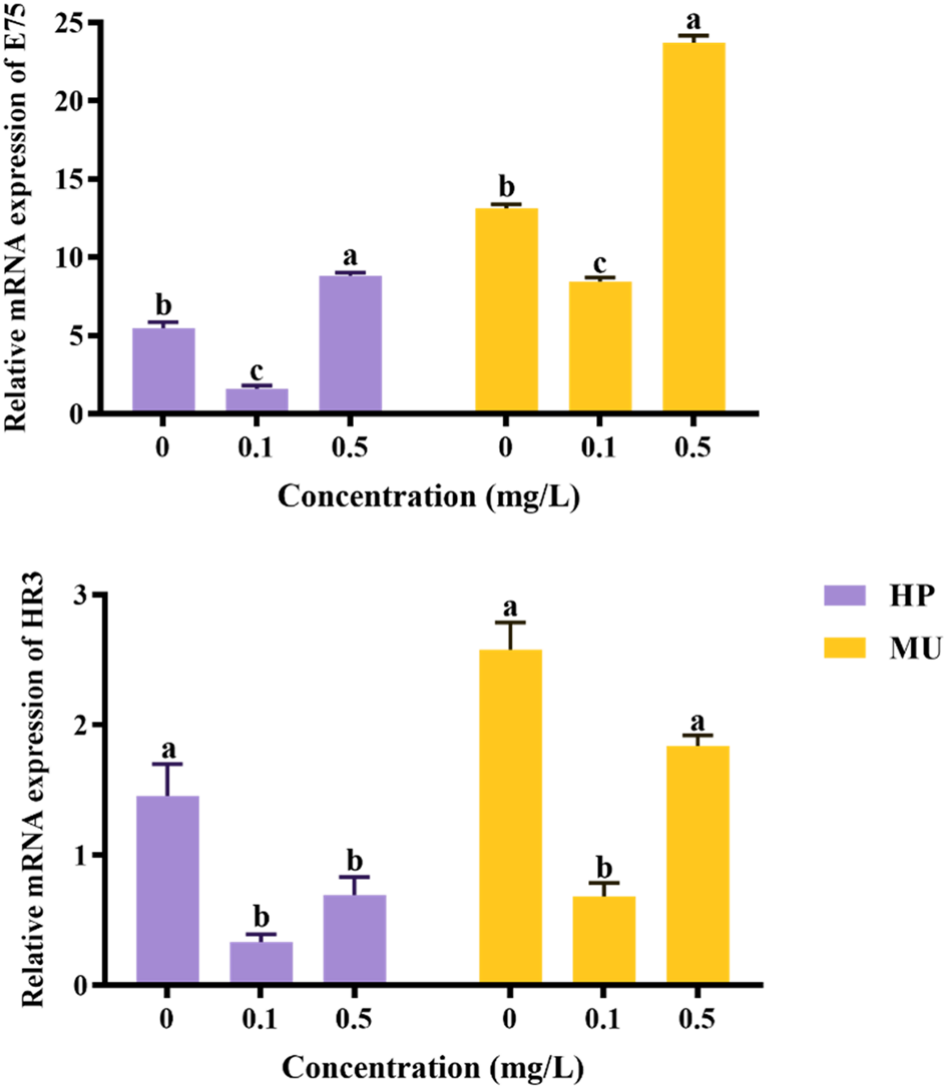
Expression level of the *E75* gene and *HR3* gene of *M. rosenbergii*. Data are presented as mean + SEM. The different letters indicate significant differences among different groups, *P* < 0.05. HP is the hepatopancreas and MU is the muscle.

The mRNA level of *E75* and *HR3* in the hepatopancreas and muscle is shown in Fig. 8. Obviously, the expression of *E75* in the hepatopancreas was significantly decreased after exposure to the 0.1 mg/L TiO_2_–GO composite nanoparticle, and significantly elevated in the 0.5 mg/L experimental group (*P* < 0.05). In addition, the mRNA expression of *E75* in the muscle was significantly increased after exposure to the 0.5 mg/L TiO_2_–GO (*P* < 0.05).

The mRNA expression of *HR3* was also notably affected by different concentrations of TiO_2_–GO nanoparticle. As shown in Fig. 8, the expression of *HR3* in the hepatopancreas was significantly decreased with the TiO_2_–GO composite nanoparticle exposure (*P* < 0.05), but there was no difference observed between the low concentration experimental group and higher group (*P* > 0.05). Additionally, after exposure to 0.1 mg/L TiO_2_–GO composite nanoparticle, the expression level of *HR3* in the muscle was significantly lower than the control group (*P* < 0.05). But there were no significant expression differences of *HR3* in the muscle between the 0.5 mg/L concentration experimental group and the control group (*P* > 0.05).

## Discussion

This study was the first time that full-length cDNAs of *E75* and *HR3* were cloned from *M. rosenbergii*. The amino acid sequences for *E75* and *HR3* were respectively determined to encode 1138 and 363 amino acid residues. The *E75* protein was deduced to contain a ZnF_C4 domain and a HOLI domain while the *HR3* protein was deduced to contain a HOLI domain. In the presence of outgroup, these two genes and their homologues were highly concordant, and the clustering results showed high affinity between the *E75* and *HR3* of some crustaceans and insects. The amino acid sequence of the *E75* protein from *M. rosenbergii* was more closely related to *Palaemon carinicauda*, and *HR3* of *M. rosenbergii* was the sister branch to the sub-cluster comprised of *Procambarus clarkii*, *Homarus americanus*, *Portumnus trituberculatus*, and *Eriocheir sinensis* within the crustacean cluster.

According to the results of expression analysis in different tissues, the highest expressions of *E75* and *HR3* were both found in muscle, followed by hepatopancreas. This result is consistent with previous studies^23^. Widespread expression of *E75* is present in many crustaceans. Combined with the wide distribution of EcR^24–26^ and RXR^27–29^ receptor heterodimers, the 20E/EcR/RXR complex acts on a variety of target tissues via *E75*, thereby mediating the ecdysis process^23^. Previous studies have shown that reduced levels of *E75* mRNA in juvenile shrimp inhibited the molting process in 96% of juvenile shrimp^30,31^, and even caused abnormal epidermal retraction and impaired development of new setae^31^.

*HR3*, an ecdysone-inducible early-late gene, was shown to be necessary for the prepupal-pupal transition and development of adult structures in *Drosophila melanogaster*^32,33^. The larvae of *Leptinotarsa decemlineata* are unable to transition into the pupal stage after silencing of *HR3*, and instead remain in the nymph form^34^. Ecdysis is dependent on rapid changes in 20-hydroxyecdysone (20E) levels in the blood, which peak when the molt happens and subsequently drop after the molt^35^. These changes control the production and release of transcription factors that control the ecdysis-related behavior^35–37^. 20E increases the expression of early genes by binding to the heterodimer of the ecdysone receptor (EcR and RXR), which subsequently upregulates the expression of the early-late gene, *HR3*^38,39^. Previous experiments have shown that after interfering with expression of *HR3* by RNAi, all 5th instar larvae failed to develop successfully into adults and died before molting. The study showed that silencing *HR3* in *Locusta migratoria* would suppress the expression of the two chitin biosynthesis genes and two chitinase genes during nymphal-nymphal and nymphal-adult transitions, and thus block the molt^39^. Based on the high levels of expression shown in muscle, there is some evidence that the *E75* and *HR3* genes are involved in the molting process of *M. rosenbergii*.

Nanomaterial exposure results show that low concentrations of TiO_2_–GO nanoparticles inhibit the expression of *E75* and *HR3*, while the relative expression of genes increases at higher concentrations. According to previous studies, inhibition at low concentrations (0.1 mg/L) indicated that nanomaterial exposure resulted in a slowing down of the growth process of juvenile *M. rosenbergii*^11^. In contrast, when concentrations reach a threshold, elevated expression of *E75* may cause juvenile *M. rosenbergii* to enter the molting stage when energy is insufficient. This disrupted the molting process and had a negative impact on growth. In addition, it has been shown that nanomaterials can have an adhesive effect on the surfaces of organisms, thus affecting their molting and growth processes^40^. Furthermore, the aggregate adhesion may cause other physical effects and a loss of mobility^41^. Finally, when a large number of nanoparticles accumulate on the surface of an organism in a short period of time, an increase in specific gravity and physical resistance during movement due to the coating of the biological surface start to take effect, which may result in a strong increase in energy demand^42^.

## Conclusion

In summary, we cloned the full-length cDNA sequences of *E75* and *HR3* from *M. rosenbergii* and investigated the gene expression under exposure to TiO_2_–GO nanomaterials. Sequence analysis showed that both *E75* and *HR3* contain a HOLI domain, with *E75* of *M. rosenbergii* being more closely related to *E75* of *Palaemon carinicauda* and *HR3* of *M. rosenbergii* being the sister branch to the sub-cluster comprised of *Procambarus clarkii*, *Homarus americanus*, *Portumnus trituberculatus*, and *Eriocheir sinensis* within the crustacean cluster. These two genes were expressed at the highest levels in muscle, followed by hepatopancreas. Analyses after exposure to TiO_2_–GO nanoparticles showed that the expression of these two genes decreased and then increased with increasing concentration. According to the results of this study, exposure to TiO_2_–GO nanoparticles had a negative impact on the molting and growth processes of *M. rosenbergii*. The effects of nanomaterials on the molting mechanism require further studies.

## Supplementary Information

Supplementary Information.

## Data Availability

The authors declare that all data supporting the findings of this study are available within the paper and its supplementary information file.
