# Supplementary material for: Molecular characterization and transcriptional response to TiO2–GO nanomaterial exposure of two molt-related genes in the juvenile prawn, Macrobrachium rosenbergii
Source: Sci Rep. 2023 Jun 27;13:10392. doi: 10.1038/s41598-023-37626-8 (PMC10300099; doi:10.1038/s41598-023-37626-8)
Supplement: Supplementary file 1 — Supplementary Information. [file 41598_2023_37626_MOESM1_ESM.docx]

**Supporting Information**

**Molecular characterization and transcriptional response to TiO_2_-GO nanomaterials exposure of two molt-related genes in the juvenile prawn,** ***Macrobrachium rosenbergii***

Ziqi Guo^1,2^, Likun Xu^1^, Wei Wang^1^, Wei Chen^1^, Chunyan Ma^1^, Fengying Zhang^1^, Lingbo Ma^1^, Zhiqiang Liu^1, *^, Keyi Ma^1, *^

1 Key Laboratory of East China Sea Fishery Resources Exploitation, Ministry of Agriculture and Rural Affairs, East China Sea Fishery Research Institute, Chinese Academy of Fishery Sciences, Yangpu Area, Shanghai 200090, P.R. China

2 College of Fisheries and Life Science, Shanghai Ocean University, Pudong New Area, Shanghai 201306, P.R. China

*Corresponding author: Keyi Ma and Zhiqiang Liu, East China Sea Fishery Research Institute, Chinese Academy of Fishery Sciences, No.300 Jungong Road, Yangpu Area, Shanghai 200090, P.R. China

E-mail: kyma1632022@163.com (Ma); 18817775160@163.com (Liu)

**S1 Materials and methods**

**S1.1 Real-time quantitative PCR analysis (qRT-PCR)**

Total RNA (1 µg) per sample was reverse-transcribed into cDNA using PrimeScript RT reagent Kit (Takara, Japan), and stored at -20°C until further use. qRT-PCR was performed in CFX96™ RT-PCR (BioRad, Hercules, CA, USA), using NovoStart^®^SYBR qPCR SuperMix Plus (Novoprotein, China). Three replicates were set for each gradient group, and the internal reference was set as the control group for each reaction. The reaction system for fluorescence quantitative PCR contained 2× ChamQ Universal SYBR qPCR Master Mix 10 μL, 2 μL cDNA template, and 0.25 μL each of the forward and reverse primers. After fully mixing the prepared system, the sample was centrifuged for 3 minutes. The sample in the pellet was then used in the qPCR reaction using a CFX96™ RT-PCR (BioRad, Hercules, CA, USA). The RT-qPCR conditions were as follows: 95 ℃ for 60 s, then 40 cycles of 95 ℃ for 20 s, and 60 ℃ for 60 s. The accuracy of the PCR dissolution curve was then determined in order to determine the amplified product. Relative mRNA levels of the target genes were analysed using the 2^−ΔΔCt^ method.

**Table S1** Sequence identity, GMQE, and QMEAN between genes and template

|  |  | Seq Identity | GMQE | QMEAN |
| --- | --- | --- | --- | --- |
| E75 | *Mr*-E75 | 30.67% | 0.17 | -4.82 |
|  | *Pv*-E75 | 30.82% | 0.25 | -4.53 |
|  | *Pc*-E75 | 30.79% | 0.19 | -4.01 |
| HR3 | *Mr*-HR3 | 34.73% | 0.49 | -0.84 |
|  | *Pv*-HR3 | 34.55% | 0.51 | -1.89 |
|  | *Pc*-OUTB | 34.44% | 0.36 | -1.69 |


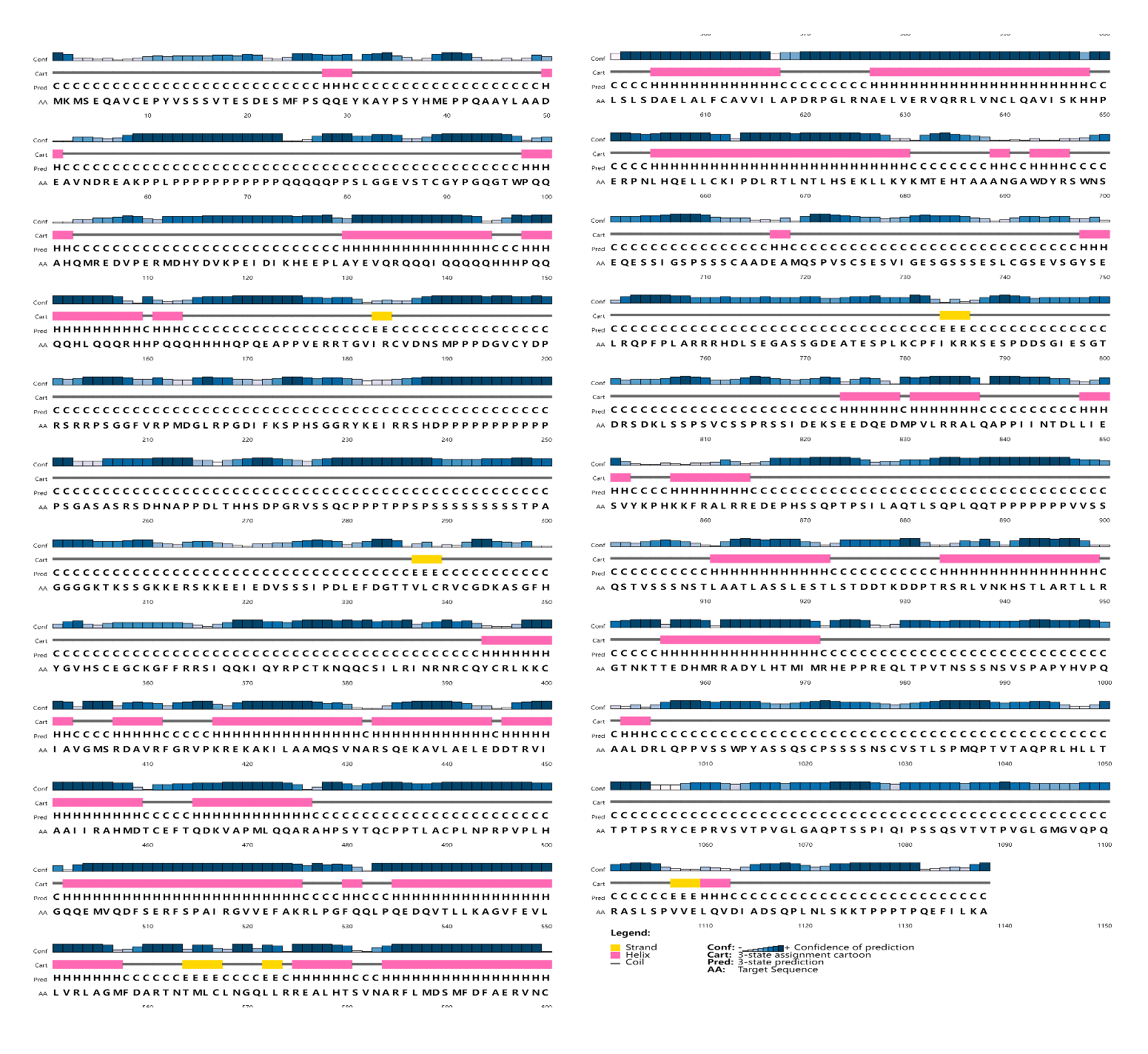


**Figure S1.** Secondary structure of the E75 protein in *Macrobrachium rosenbergii*


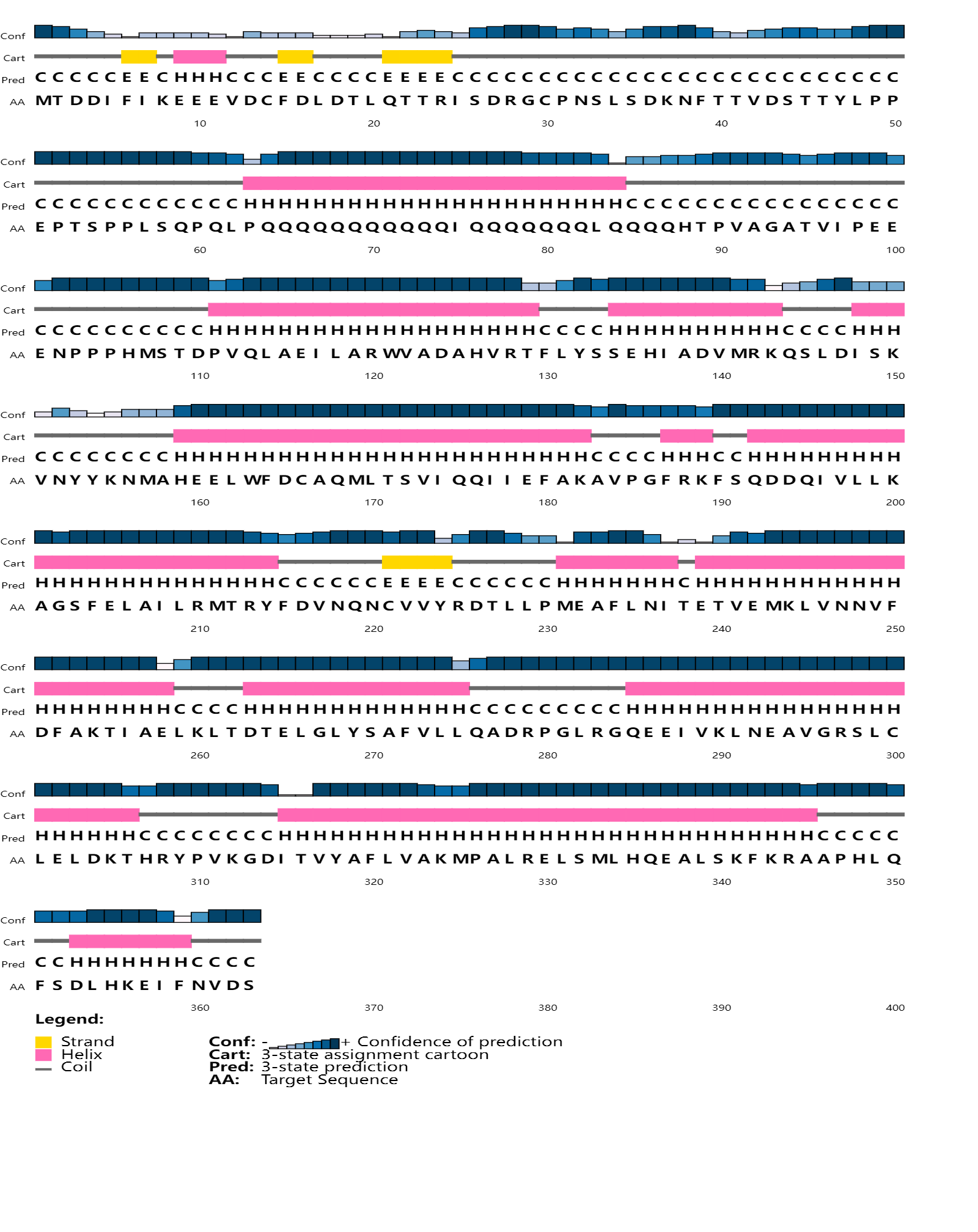


**Figure S2.** Secondary structure of the HR3 protein in *Macrobrachium rosenbergii*
